# Supplementary material for: Theory of Food: Unravelling the Lifelong Impact of Childhood Dietary Habits on Adult Food Preferences across Different Diet Groups
Source: Nutrients. 2024 Jan 31;16(3):428. doi: 10.3390/nu16030428 (PMC10857510; doi:10.3390/nu16030428)
Supplement: Supplementary file 1 [file nutrients-16-00428-s001.zip › nutrients-2816501-supplementary.pdf]

Validation of
the Childhood Food Preferences Questionnaire (CFPQ) and the Adulthood Food Preferences Paradigm (AFPP)

The Childhood Food Preferences Questionnaire (CFPQ) validation process was executed meticulously to ensure the reliability and credibility of the collected data. Content validation involved a comprehensive dietician review and survey methodology, confirming that the questionnaire effectively covered all relevant subject aspects. A pilot test was conducted with a representative sample (n=40) from the target population, revealing valuable insights into potential ambiguities or issues with question wording. The questionnaire underwent refinement for improved clarity and precision. Test-retest reliability measures were employed to assess the consistency of responses over time, further validating the questionnaire's stability. The culmination of these validation steps assures that the CFPQ is a valid instrument capable of accurately capturing information on the frequency of food consumption during the subject's childhood within the intended population.

In this test-retest reliability assessment, we aimed to investigate the consistency of the Childhood Food Preferences Questionnaire (CFPQ) over a specified time frame. The assessment was administered twice to a sample of young, healthy adults (n=40), with a time interval of three weeks between administrations. The primary objective was to measure the stability of scores over time, examining the extent to which individuals' responses remained consistent across the two test sessions. This time frame was chosen to balance capturing potential fluctuations in the measured construct and minimizing the likelihood of recall bias. The Test-retest reliability analysis revealed a valid Pearson correlation coefficient between scores obtained in the first and second administrations ($r_p(38) = .78$, $p = .003$).

In the validation process of the computerized **Adulthood Food Preferences Paradigm (AFPP)**, rigorous measures were implemented to ensure the reliability and accuracy of the collected data. Firstly, the task underwent expert review by nutritionists and psychologists to ascertain its alignment with established dietary guidelines and psychological constructs related to eating behavior. Subsequently, a pilot study (n=17) was conducted with diverse participants to identify potential ambiguities or misconceptions in task instructions. Adjustments were made based on the feedback received, and the finalized task was then administered to a representative sample of the target population (n=35). Participants' responses were cross-verified during data collection with traditional dietary assessments, such as food diaries and 24-hour recalls, to validate the computerized task's outcomes.

Statistical analyses, including correlation and reliability assessments, were employed to establish the task's consistency and validity in measuring food consumption patterns in adulthood. The comprehensive validation process ensured the robustness of the computerized task and bolstered the credibility of the study's findings.

In this test-retest reliability assessment, we aimed to investigate the Adulthood Food Preferences Paradigm (AFPP) consistency over a specified time frame. The assessment was administered twice to a sample of young, healthy adults (n=35), with a time interval of two weeks between administrations. The primary objective was to measure the stability of scores over time, examining the extent to which individuals' responses remained consistent across the two test sessions. This time frame was chosen to balance capturing potential fluctuations in the measured construct and minimizing the likelihood of recall bias. The Test-retest reliability analysis revealed a valid Pearson correlation coefficient between scores obtained in the first and second administrations ($rp(33) = .71$, $p = .005$). In addition, we also calculated the association of the AFPP with the Israeli-Mediterranean diet assessment tool ($rp(33) = 0.67$, $p = 0.05$) and their self-reports.
